# Signature Definition Shapes Associations Between Exenatide-Related Transcriptional Programs and Macrophage Senescence Scores in Colorectal Cancer

**DOI:** 10.3390/genes17101266

**Published:** 2026-10-12

**Authors:** Yunying Song, Aliya Tayier, Yu Peng, Tiankang Li, Lijun Xu, Minhui Zhong, Chan Yu, He Ma

**Affiliations:** 1 Shanghai Key Laboratory of Maternal Fetal Medicine, Department of Intensive Care Unit, Shanghai Institute of Maternal-Fetal Medicine and Gynecologic Oncology, Shanghai First Maternity and Infant Hospital, School of Medicine, Tongji University, Shanghai 200092, China; songyunying@51mch.com; 2 Shanghai Key Laboratory of Maternal Fetal Medicine, Department of Breast Surgery, Shanghai Institute of Maternal-Fetal Medicine and Gynecologic Oncology, Shanghai First Maternity and Infant Hospital, School of Medicine, Tongji University, Shanghai 200092, China; 2511261@tongji.edu.cn (A.T.); 2412017@tongji.edu.cn (T.L.);; 3 Shanghai Key Laboratory of Maternal Fetal Medicine, Department of Gynecology, Shanghai Institute of Maternal-Fetal Medicine and Gynecologic Oncology, Shanghai First Maternity and Infant Hospital, School of Medicine, Tongji University, Shanghai 200092, China; 2432495@tongji.edu.cn; 4 Department of Neurology, Shanghai East Hospital, School of Medicine, Tongji University, Shanghai 200120, China; lijun_xu0921@163.com

**Keywords:** exenatide, colorectal cancer, macrophages, senescence signatures, transcriptomics, single-cell RNA sequencing, virtual perturbation, sensitivity analysis

## Abstract

**Background:** Treatment-derived expression signatures are often applied to untreated tumor cohorts, although their biological meaning may depend on signature construction and cellular composition. We examined the relationship between an exenatide-derived signature and senescence-associated macrophage expression in colorectal cancer. **Methods:** Archived pan-cancer and patient-level results were examined alongside a reconstruction from public mouse bulk RNA sequencing and colorectal single-cell matrices. Exenatide signatures were re-derived with tissue and batch adjustment, within-mouse correlation, and one-to-one orthologue mapping. Shared-gene removal, stricter macrophage annotation, expression-matched random signatures, virtual perturbation summaries, and three murine treatment comparisons assessed interpretation and robustness. **Results:** The original signature was associated with SenMayo in stomach, colon, and rectal tumor analyses. Reconstruction retained 209,207 colorectal cells and 4316 tumor macrophages, with 17 patients eligible for tumor-level analysis. Removing CXCR2 and IL1B from both signatures preserved a positive association (Spearman ρ = 0.804; adjusted β = 1.526; *p* = 0.00219). The corresponding mean expression association exceeded most expression-matched random signatures (empirical *p* = 0.006). Associations persisted with a blocked log-expression derivation but were absent with a blocked voom-derived signature using one-to-one orthologues (ρ = 0.056; *p* = 0.620). Archived virtual IL1B deletion and overexpression predicted opposing shifts after multiplicity adjustment. Treatment–macrophage fold-change correlations remained small (ρ = −0.038 to 0.046), with heterogeneous enrichment. The association was attenuated after adjustment for either inflammatory score—it remained statistically detectable after TNFα/NF-κB adjustment (β = 0.672, 95% CI 0.307–1.037) and was less precisely estimated after broader inflammatory response adjustment (β = 0.383, 95% CI −0.031–0.797). **Conclusions:** The original exenatide-derived signature was associated with SenMayo scores in colorectal tumor macrophages after removal of shared genes. This association overlapped with inflammatory expression and varied with signature derivation. Dependence on signature derivation limits its interpretation as a pharmacological response marker. These findings support investigation of inflammatory macrophage states without establishing drug-induced senescence.

## 1. Introduction

Glucagon-like peptide-1 receptor agonists alter transcriptional programs across tissues. In aged mice, exenatide has been studied using functional measurements and multi-tissue molecular profiling [1]. Applying a signature from such an experiment to human tumors asks whether tumor expression resembles that treatment-associated pattern. It does not establish treatment exposure, receptor engagement, or a corresponding drug effect in the tumor. GLP-1 receptor signaling also engages innate immune cells: exendin-4 attenuates lipopolysaccharide-induced inflammatory activation in macrophages [2], exenatide suppressed chemically induced colon carcinogenesis in diabetic mice [3], and observational studies have also examined associations between GLP-1 receptor agonist use and colorectal cancer risk [4]. Separately, senescent macrophage states have been described in tumors [5], and in colorectal cancer macrophage-derived IL1B can induce senescence in cancer-associated fibroblasts and promote chemoresistance [6]. These links motivate asking whether an exenatide-associated transcriptional pattern intersects senescence-associated macrophage programs in colorectal cancer; they do not, by themselves, imply a drug effect on tumor senescence.

Senescence-associated gene sets offer a complementary description of tumor states. SenMayo and the Fridman signature capture aspects of senescence-related expression, but their genes can also participate in inflammatory activation and tissue remodeling [7,8]. This overlap is particularly relevant to macrophages. An association between a treatment-derived score and a senescence score may reflect coordinated biology, shared genes, cellular composition, or choices made during signature derivation.

We investigated these possibilities for a multi-tissue exenatide signature. Initial pan-cancer analyses identified gastrointestinal associations and a prominent colorectal macrophage signal. We then reconstructed the treatment signature and colorectal single-cell analysis from public data, removed shared genes, and examined alternative treatment models and orthologue mappings. Virtual perturbation predictions and independently recomputed murine treatment comparisons provided additional context. The study addresses the reproducibility and interpretation of a transcriptional association, rather than efficacy or causality of treatment.

## 2. Materials and Methods

### 2.1. Study Design and Data Sources

Two analysis representations were distinguished(Table 1). The initial discovery analysis used Toil-recomputed TCGA and GTEx expression across 13 cancer types and an original preprocessing of GSE294300 colorectal single-cell data [9]. Available patient-level scores and regression tables were retained to reproduce these observations. The subsequent reconstruction started from public GSE294300 matrices and the depositor’s retained-cell list. Both single-cell representations concern the same 18 patients and are processing sensitivity analyses, not independent clinical cohorts.

GSE280382 AgedLT supplied the exenatide signature. Exact matching between count columns and sample metadata selected 93 treated tissue samples from 8 mice and 102 control tissue samples from 9 mice, spanning 12 tissues. Repeated tissues were, therefore, distinguished from independent animals. The public sample metadata and the provenance of these assignments are given in Supplementary Table S1b,c. GSE302656 comprised five liraglutide-treated and five control lung tumors in obese mice. Its count-column library identifiers were matched directly to GEO sample descriptions: RS-04402142 through RS-04402146 were controls, and RS-04402147 through RS-04402151 were treated samples. GSE338091 contained five control and five semaglutide-treated aged-mouse liver samples. Human treatment exposure was unavailable.

### 2.2. Reconstruction and Re-Derivation of the Exenatide Signature

The original definition was reproduced using edgeR expression filtering and TMM normalization, log-counts per million with a prior count of 1, and limma modeling of treatment and tissue [10,11]. The original filtered object retained the pre-filter library sizes. Significant genes required Benjamini–Hochberg FDR < 0.05 and absolute log2 fold change > 0.5. MGI mouse-to-human mapping reproduced the original first-match rule. This recovered 24 upregulated and 7 downregulated human genes. The exact reconstructed gene memberships and their provenance are provided in Supplementary Table S2a–c.

Alternative derivations used a filtered-count object with recalculated library sizes and TMM normalization. One analysis fitted log-expression with tissue and batch covariates, estimated within-mouse correlation using duplicateCorrelation, and used robust trend empirical Bayes moderation. A second used a two-pass voom procedure: initial precision weights and within-mouse correlation were estimated, weights were recalculated with that correlation, and the final blocked model was fitted with tissue and batch adjustment and robust empirical Bayes moderation [10]. The treatment coefficient represented the average contrast under an additive tissue model. Tissue-specific treatment effects were not assumed identical biologically.

Each derivation was evaluated with first-match and strictly one-to-one mouse–human mappings. One-to-one mapping retained pairs for which neither symbol mapped to multiple partners. The blocked voom model with one-to-one mapping defined the principal re-derived comparison signature. These alternative analyses were post hoc sensitivity analyses—none was selected according to its association with the human senescence scores. Gene membership counts for every derivation are summarized in Supplementary Table S10a, and the estimated within-mouse correlations used by the blocked models are given in Supplementary Table S10b. All gene-level outputs and gene membership files are supplied.

SenMayo was reconstructed from the original human supplementary table [7], and Fridman from MSigDB [8]. Their 125-gene and 77-gene GMT files matched the archived checksums. The 59-gene union of the two original Reactome GLP-1 sets was also reconstructed from msigdbr 7.5.1, although receptor and downstream scores were not treated as substitutes for the treatment-derived score.

### 2.3. Archived Bulk and Patient-Level Analyses

Bulk scores used single-sample gene set enrichment analysis, with the response score defined as the upregulated-set score minus the downregulated-set score [12]. Available SenMayo regression models included age, stage, ABSOLUTE purity, and MCP-counter fibroblast and monocytic-lineage estimates where available [13,14]. Twelve complete-case cohort models formed the reported bulk FDR family. Their original coefficient and standard error tables are provided in Supplementary Table S3a. Two further archived outputs were retained for completeness without being used to select the colorectal focus: response-score survival models (Supplementary Table S3b) and descriptive correlations among the receptor, response, and downstream measurements across 15 tumor and normal-tissue strata (Supplementary Table S3c).

The original colorectal preprocessing used EmptyDrops cell calling, feature and mitochondrial quality filters, per-sample cell caps, doublet removal, normalization, principal components, and marker-based annotation [15]. Harmony integration was used for visualization [16]. It contained 278,083 cells and 8240 tumor macrophages. Original Seurat module scores were averaged within patient, tissue, and lineage groups with at least 20 cells. Tumor models adjusted for age and sex, with BH correction across eligible lineages within each senescence signature. Patient influence was assessed by omitting each tumor patient in turn. For complete tumor–normal pairs, regression on score differences reproduced the coefficient from the tissue- and patient-fixed-effects model. Bootstrap confidence intervals used 10,000 patient resamples. These calculations used the supplied patient score table (Supplementary Table S4), with reproduced lineage models in Supplementary Table S6 and patient influence, paired-regression, and bootstrap checks in Supplementary Table S8a–c. They did not treat individual cells as independent patients.

### 2.4. Reconstruction of Colorectal Single-Cell Scores

All 36 public GSE294300 matrices were read independently. Cells were selected using the depositor’s 214,854-cell list, matching nucleotide barcodes within each sample after removing the merged-object suffix. Duplicate gene symbols were summed. Cells with more than 200 and fewer than 6000 detected genes and a mitochondrial fraction below 20% were retained. Counts were normalized to 10,000 per cell and log1p transformed. No additional cell calling, random cell cap, or new doublet classifier was applied to this depositor-filtered representation. Per-sample reconstructed counts and quality metrics are given in Supplementary Table S11a.

Lineage assignment used the largest mean normalized expression among nine fixed marker panels. Myeloid cells were divided into macrophages or neutrophils by comparing C1QA, C1QB, CD68, APOE, and MSR1 with CSF3R, S100A8, S100A12, FPR1, and CXCR2. A stricter sensitivity definition required at least two detected macrophage markers and a margin of 0.1 above the next-best lineage score. Marker panels, cell assignments, and per-sample counts are provided with the code. This transparent rule-based reconstruction was not assumed to reproduce the original clustering exactly.

Expression-matched control-adjusted scores were calculated for each program component. Genes were divided into 24 bins using mean expression across all reconstructed cells. For each target gene, 100 controls were sampled from its expression bin, expanding to an adjacent bin if needed. Every study-signature gene was excluded from the control pool, and control genes were kept separate between distinct program components. Sampled controls were deduplicated within a component. The score was the mean target expression minus the mean control expression, and response scores were the up-component score minus the down-component score. Random sampling used seed 42. The complete control lists are supplied, so score reconstruction does not depend on regenerating a random draw.

CXCR2 and IL1B were shared by the original response signature and SenMayo. Both were removed from both programs for the shared-gene sensitivity analysis, retaining the original controls. The principal re-derived signature shared no genes with SenMayo. Unadjusted mean expression scores were also evaluated to test dependence on control subtraction.

Scores were aggregated before inference. Patient–tissue groups required at least 20 macrophages. Tumor models adjusted for age and sex, and additional models included mean log-library size and mitochondrial fraction. Paired analyses used within-patient tumor-minus-normal differences. BH adjustment covered 48 distinct reconstruction models across score methods, annotation definitions, outcomes, and adjustment sets. Identical shared-gene-removal models were omitted when the signatures already had no overlap. Reconstructed patient scores and the full set of 48 distinct models are given in Supplementary Table S11b,c, and gene coverage per program component in Supplementary Table S11d.

As an additional specificity check, 1000 random response signatures matched the expression bins and up/down sizes of the disjoint original signature. All study-signature genes were excluded from candidate controls. Mean expression scores were correlated with disjoint SenMayo across the same tumor patients. The one-sided empirical *p*-value was calculated as one plus the number of random correlations at least as large as the observed correlation, divided by 1001. Individual random-signature correlations are given in Supplementary Table S13b and their distribution in Supplementary Figure S1. This tests performance relative to expression-matched random genes, not specificity for senescence over every inflammatory program. Inflammation-adjustment analyses additionally scored the MSigDB Hallmark inflammatory response and TNFα-signaling-via-NF-κB gene sets (200 genes each) as mean log-normalized expression per patient, correlated these scores with SenMayo and the response signature, and entered them as additional covariates in age- and sex-adjusted models, each model using its own residual degrees of freedom (Supplementary Table S14a,b). The inflammation scores were additionally recomputed after removing all genes shared with SenMayo or the treatment signature (union-removed; 172 and 173 candidate genes) and entered in the same models. For the across-gene treatment correlations, the pseudobulk quasi-likelihood model was refitted, leaving out each patient in turn, and the Spearman correlations with each treatment ranking were recomputed from each refit (Supplementary Table S15). For tissue composition sensitivity, the legacy signature derivation was refitted after excluding each of the twelve tissues in turn, and each leave-one-tissue-out signature was scored in the same patients, with the fold-specific SenMayo-overlapping genes removed from both the signature and the SenMayo score in a separate pass (Supplementary Table S16a). Tissue-specific treatment effects for the original signature genes were estimated from a cell-means model with tissue-by-group contrasts (Supplementary Table S16b).

### 2.5. Virtual Perturbation Predictions

The original Geneformer V2-104M analysis used 2000 stratified macrophages: 530 high-score cells, 668 low-score cells, and 802 intermediate cells [17]. High- and low-state centroids were derived from the extreme groups, and perturbations were simulated in intermediate cells. These groups were disjoint at the cell level but arose from the same patients. The readout was the change in cosine similarity to the high-state centroid minus the change toward the low-state centroid.

IL1B and CDKN1A deletion and overexpression and GLP1R overexpression were summarized using patient median shifts. Deletion required presence of the target token and, therefore, involved fewer cells than overexpression. We applied a post hoc BH correction across the five available patient-level Wilcoxon *p*-values. Aggregate predictions for the five perturbations are given in Supplementary Table S5 and the multiplicity correction in Supplementary Table S9. These predictions remain conditional on the original state labels and were not regarded as validation of the re-derived signature. Benchmarking studies reporting task-dependent limitations of single-cell foundation models informed interpretation [18,19].

### 2.6. Recomputed Treatment Comparisons

Counts from reconstructed tumor macrophages were summed by patient. Eligible patients were divided at their median SenMayo score into eight high-score and nine low-score patients. Differential expression used an age- and sex-adjusted edgeR quasi-likelihood model with robust dispersion estimation [11]. Treatment analyses for liraglutide and semaglutide used their verified group labels (liraglutide library-to-group assignments in Supplementary Table S12a) and the same quasi-likelihood framework. The exenatide comparison used the blocked voom model described above. Mouse–human comparisons used one-to-one orthologues without duplicated human gene rows.

Macrophage genes meeting FDR < 0.05 were separated by fold-change direction into high-state upregulated and downregulated sets, and the complete pseudobulk differential expression table is given in Supplementary Table S12b. Each set and SenMayo were tested in the three treatment-ranked lists. Rankings used the signed square root of the quasi-likelihood F statistic or the moderated t statistic. GSEApy multilevel preranked enrichment used seed 42, sample size 101, minimum set size 10, and an estimation floor of 10^−10^ [20]. BH adjustment covered all nine treatment–gene set tests. Across-gene Spearman correlations and direction-specific enrichment results are given in Supplementary Table S7a,b. Their *p*-values are descriptive because genes are dependent and do not represent independent patients.

### 2.7. Software and Reporting

Reanalyses used R 4.3.3, edgeR 4.0.16, limma 3.58.1, and statmod 1.5.0, together with Python 3.12.14, NumPy 2.3.5, pandas 2.2.3, SciPy 1.17.0, and Matplotlib 3.10.8, and GSEApy 1.3.1. Exact versions, source URLs, sample maps, checksums, gene lists, code, and numerical outputs are included in the Supplementary_Data, Supplementary_Tables and Figure S1. Original archived models and newly reconstructed analyses are identified separately. Confidence intervals are 95%, secondary analyses are exploratory, and no effect was interpreted solely through its *p*-value.

## 3. Results

### 3.1. The Original Signature Identifies Gastrointestinal and Macrophage Associations

**Figure 1:**
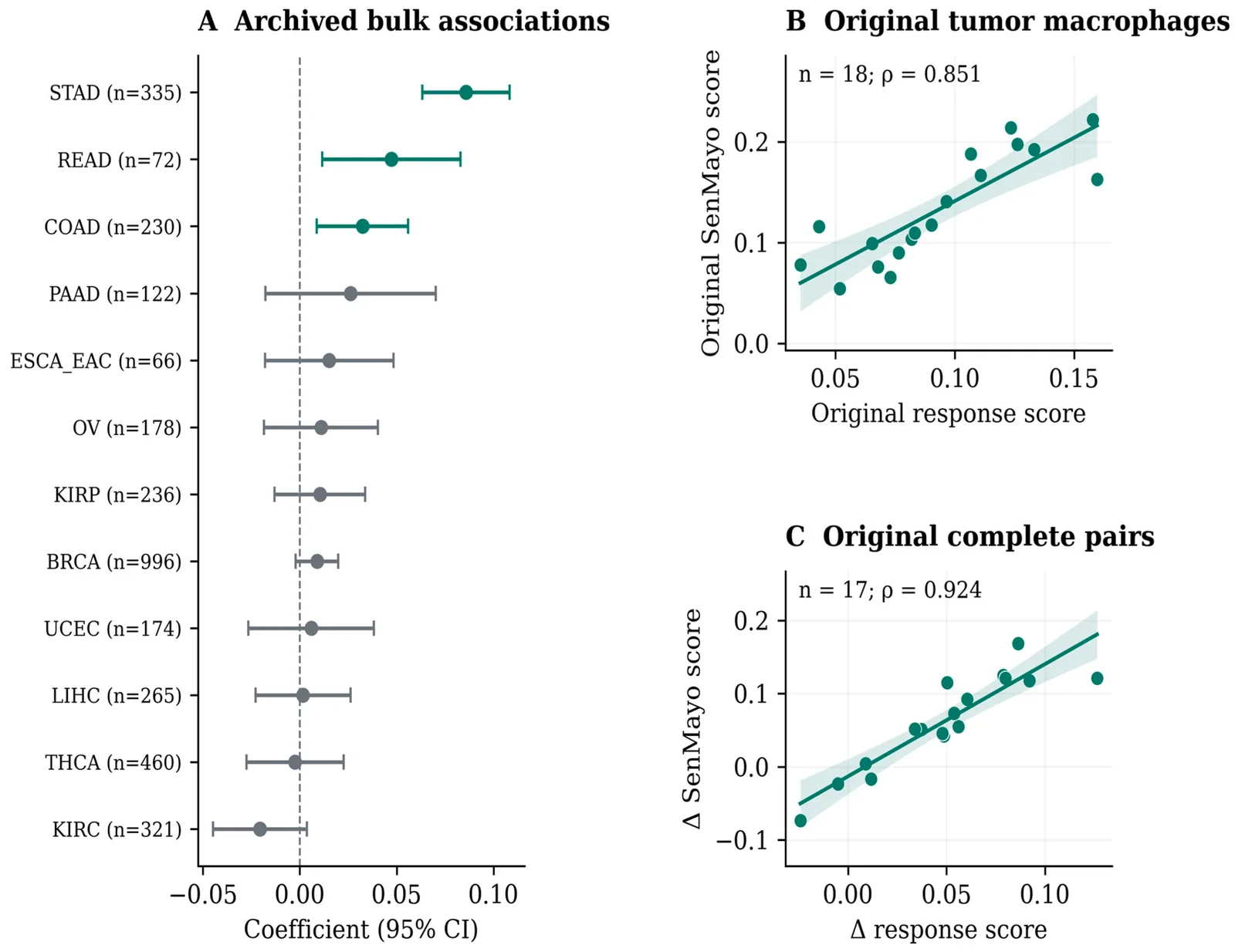
Original discovery associations. (**A**) SenMayo coefficients from the 12 available composition-adjusted bulk models: intervals are β ± 1.96 × SE, and green indicates BH FDR < 0.05 within that family. (**B**) Original tumor macrophage scores in 18 patients. (**C**) Tumor-minus-normal changes in 17 complete pairs. Each scatter point is one patient. Lines show least-squares fits with 95% confidence bands. These archived score representations are distinct from the public-cell-list reconstruction in Figure 2—both concern the same colorectal cohort.

The original signature showed positive composition-adjusted SenMayo associations in STAD, COAD, and READ among 12 evaluable bulk cohorts (Figure 1A). Coefficients were 0.0857, 0.0324, and 0.0473, respectively, with FDR values of 1.05 × 10^−11^, 0.045, and 0.047 and sample sizes of 335, 230, and 72. The same FDR family also included breast invasive carcinoma, which showed a positive but non-significant coefficient (BRCA; β = 0.0089; *n* = 996; nominal *p* = 0.113; FDR = 0.271; Figure 1A). Breast was retained in the archived family but was not carried forward, because the colorectal analyses were motivated by the significant gastrointestinal associations and the macrophage signal described below. Descriptive score correlations for the full and postmenopausal breast subsets, alongside the other strata, are reported in Supplementary Table S3c. These observations motivated the colorectal macrophage analysis—they do not establish a tumor response to exenatide.

**Figure 2:**
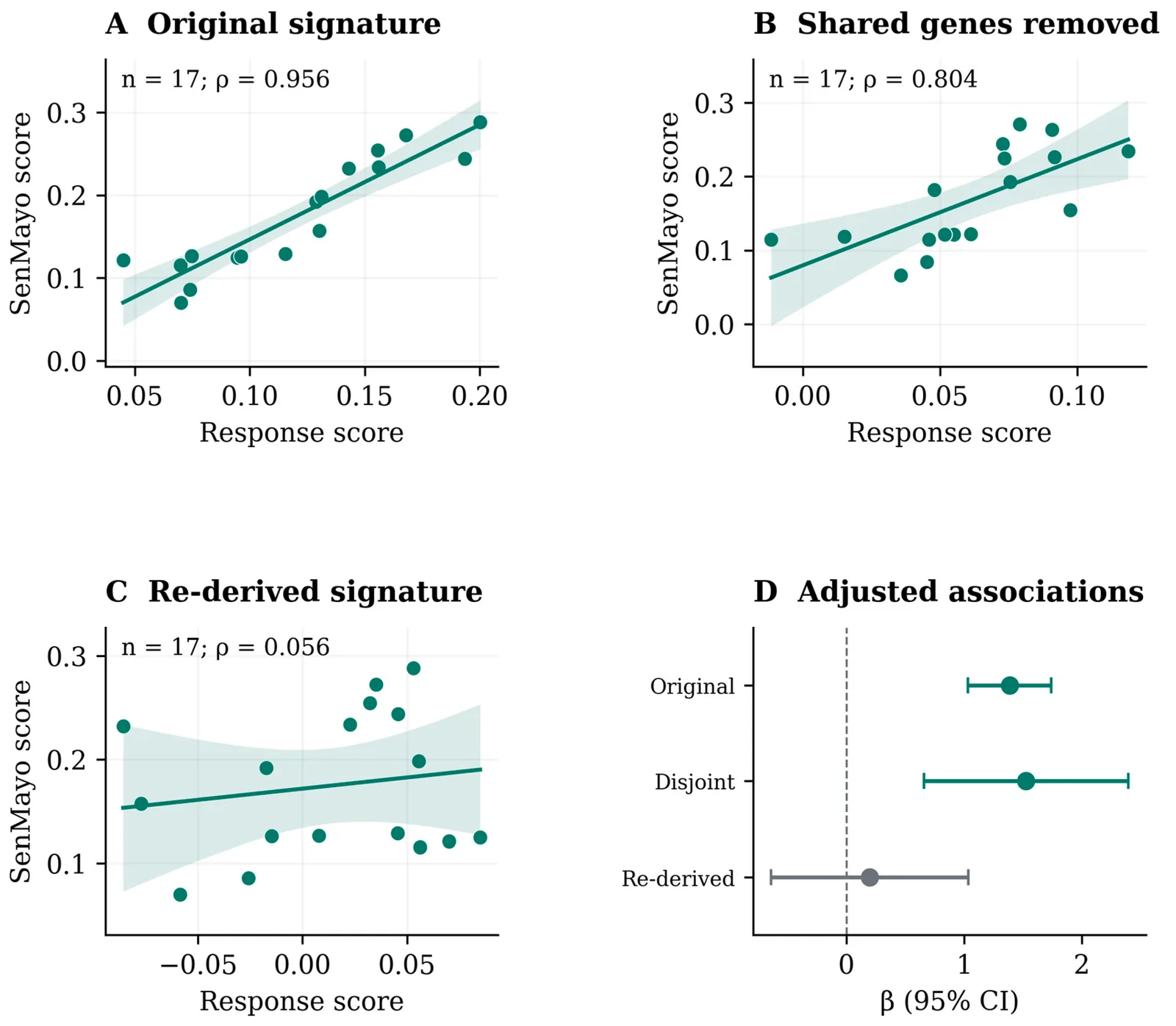
Reconstructed tumor macrophage associations. (**A**) Original response signature and SenMayo. (**B**) Both programs after removal of CXCR2 and IL1B. (**C**) The blocked voom-derived one-to-one signature and SenMayo. Scores use fixed, separately assigned expression-matched control genes. Each point represents 1 of 17 patients with at least 20 tumor macrophages under the reconstructed annotation, and lines show least-squares fits with 95% confidence bands. (**D**) Age- and sex-adjusted coefficients with t-based 95% confidence intervals for the three comparisons. Coefficients depend on the score definition and should not be interpreted as standardized biological effect sizes. Teal indicates estimates and fits of the original analyses; gray indicates the re-derived variant (panel (**D**)).

In the original patient score table, tumor macrophage response and SenMayo scores correlated across 18 patients (ρ = 0.851; Figure 1B). The age- and sex-adjusted coefficient was 1.289 (95% CI 0.829–1.748; *p* = 3.17 × 10^−5^; lineage-family FDR = 0.000317). Omitting one patient at a time yielded correlations of 0.824–0.900 and coefficients of 1.186–1.491 (Supplementary Table S8a). Across 17 complete pairs, score changes correlated at ρ = 0.924, and the paired regression coefficient was 1.536 (*p* = 4.47 × 10^−7^; Figure 1C; Supplementary Table S8b), with bootstrap intervals in Supplementary Table S8c. These results show that the association is numerically stable within the original representation.

### 3.2. Treatment Signature Membership Depends on the Derivation Model

Reproducing the original library-size handling and model recovered the 24-up and 7-down signature. Recalculating library sizes after filtering, while retaining an unblocked log-expression model, changed this to 23 upregulated and 8 downregulated genes. IL1B lay near the original fold-change selection threshold. This illustrates that a stable total gene count can conceal a change in membership.

**Figure 3:**
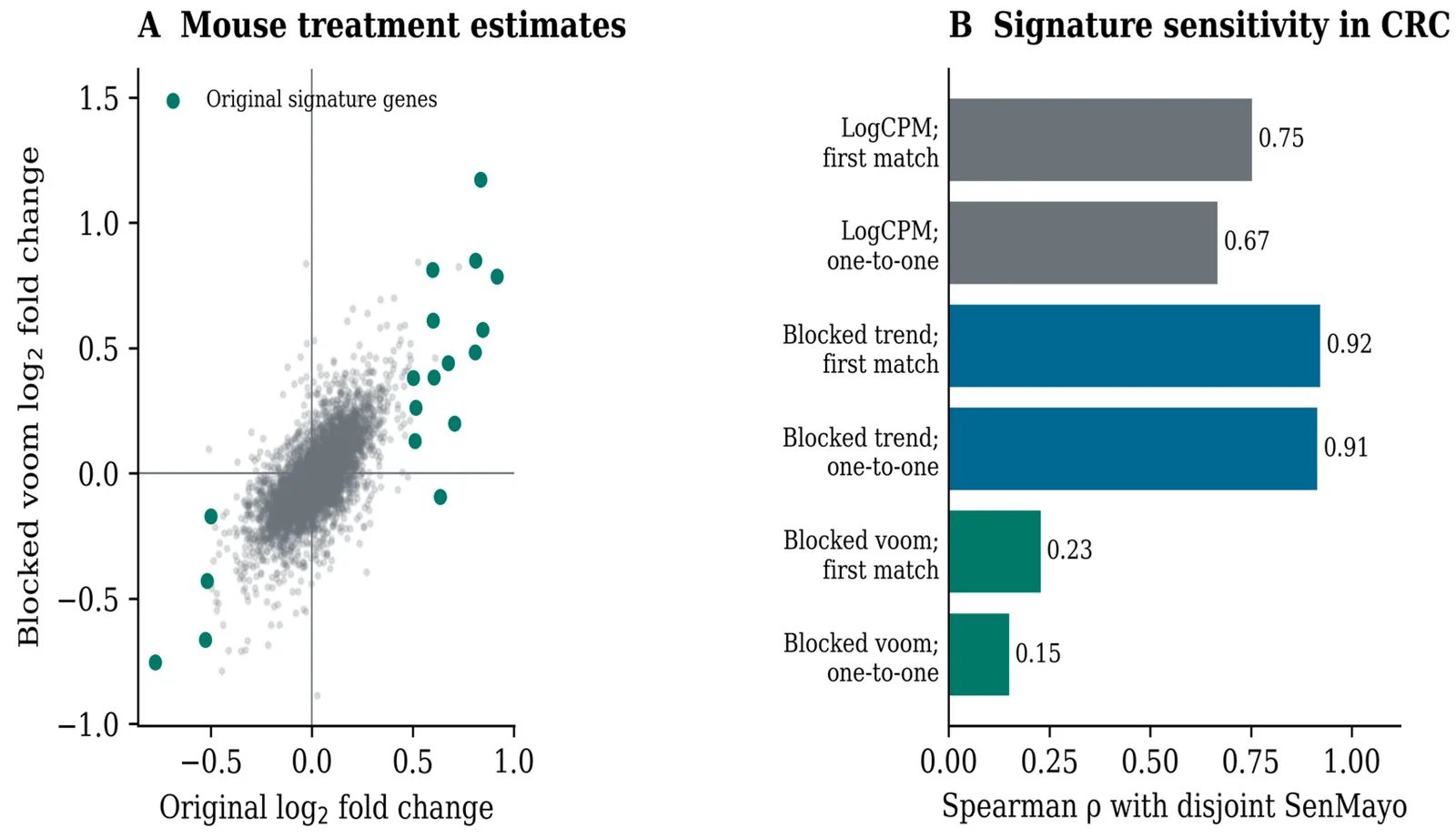
Sensitivity to treatment signature derivation. (**A**) Original and blocked voom mouse treatment log2 fold changes for one-to-one mapped genes—highlighted genes met the original signature thresholds. (**B**) Correlations of six alternative signatures with disjoint SenMayo mean expression scores in 17 reconstructed tumor macrophage samples. LogCPM indicates the sensitivity model with library sizes recalculated after filtering. Blocked trend and blocked voom models include tissue, batch, and within-mouse correlation. The figure separates first-match from one-to-one orthologue mapping. These comparisons change model assumptions and signature membership—they do not isolate a single cause of the difference.

Accounting for tissue, batch, and within-mouse dependence also changed the selected genes. The blocked trend model yielded 20 upregulated and 7 downregulated genes with first-match mapping, whereas the blocked voom model yielded 19 and 20. Restricting the latter to one-to-one orthologues produced a 15-up and 12-down signature. Estimated consensus within-mouse correlations were approximately 0.022 (Supplementary Table S10b). The derivation comparison, therefore, involves normalization, variance modeling, and mapping, as well as repeated sampling—the changes cannot be attributed to mouse dependence alone (Figure 3; Supplementary Table S10a,b).

When evaluated using mean expression scores in reconstructed tumor macrophages, signatures from blocked trend models retained positive correlations with disjoint SenMayo (ρ = 0.922 with first-match mapping and 0.914 with one-to-one mapping). Corresponding blocked voom signatures gave smaller, non-significant associations (ρ = 0.228 and 0.150; nominal regression *p* = 0.368 and 0.570). Thus, the human association was sensitive to the method used to derive the treatment signature rather than uniformly preserved after re-derivation. All six derivation–mapping combinations are tabulated in Supplementary Table S13a.

### 3.3. Shared-Gene Removal Preserves the Original Association in Reconstructed Macrophages

Public-data reconstruction retained 209,207 cells, including 103,872 tumor cells and 105,335 adjacent-normal cells. Marker assignment identified 5089 macrophages, of which 4316 were from tumors. Seventeen patients had at least twenty tumor macrophages, and ten had evaluable macrophage pairs under this reconstruction (Supplementary Table S11a,b). The different cell and pair counts reflect the depositor-based cell selection and explicit annotation rules.

The original response score correlated with SenMayo across the 17 tumor patients (ρ = 0.956; adjusted β = 1.386, 95% CI 1.032–1.741; Figure 2A). Removing both shared genes, CXCR2 and IL1B, preserved the association (ρ = 0.804; β = 1.526, 95% CI 0.659–2.393; *p* = 0.00219; reconstruction-family q = 0.00943; Figure 2B,D). Additional library-size and mitochondrial adjustment gave β = 1.318 and *p* = 0.0138 (q = 0.0332). Under the stricter macrophage definition, the disjoint association remained positive in 16 patients (ρ = 0.797; *p* = 0.00424). All 48 distinct reconstruction models are given in Supplementary Table S11c.

The disjoint mean expression score also correlated with SenMayo (ρ = 0.752). Its correlation exceeded that of most expression-matched random signatures (empirical *p* = 0.006; Supplementary Figure S1; Supplementary Table S13b). These findings show that the original tumor-level association is not explained solely by its two shared genes or an arbitrary expression-matched gene list. The disjoint paired-difference analysis was less certain in ten pairs (ρ = 0.600; *p* = 0.234), so the shared-gene-independent result was not established for within-patient changes. Inflammatory activation tracked SenMayo closely in these macrophages (Spearman ρ = 0.963 and 0.914 between the full Hallmark scores and the full SenMayo score). In the mean expression analysis, the association was attenuated after adjustment for either inflammatory score: it remained statistically detectable after TNFα/NF-κB adjustment (β = 0.672, 95% CI 0.307–1.037), whereas the estimate after broader inflammatory response adjustment was less precise (β = 0.383, 95% CI −0.031–0.797). Because the Hallmark sets share 25 and 27 genes with SenMayo, the analysis was repeated with disjointed inflammation scores (172 and 173 genes after removing all genes shared with SenMayo or the treatment signature): the association was again attenuated, remaining detectable after TNFα/NF-κB adjustment (β = 0.856, 95% CI 0.438–1.275) and less precisely estimated after inflammatory response adjustment (β = 0.447, 95% CI −0.054–0.948). These analyses quantify a substantial inflammatory component shared with senescence scoring that is not reducible to direct gene overlap (Supplementary Table S14a,b). The full-data refit under this pipeline reproduced the packaged main-text signature exactly (24 upregulated and 7 downregulated genes; ρ = 0.929 with the full SenMayo score and 0.752 after two-sided removal of the shared genes). Leave-one-tissue-out re-derivations kept signature sizes in a similar range (20–29 upregulated and 6–13 downregulated genes) and retained 75–100% of the original upregulated list, yet produced macrophage associations ranging from ρ = −0.002 to 0.922 against the full SenMayo score (seven of twelve refits *p* < 0.05) and from −0.466 to 0.757 after two-sided removal of each fold’s SenMayo-overlapping genes (four of twelve refits *p* < 0.05). Excluding colon alone gave ρ = 0.591 and −0.341 under the two scorings. Mean effects of the upregulated and downregulated gene sets had the expected directions across tissues, while individual-gene effects varied by tissue: mean log fold changes of the upregulated genes were positive in all twelve tissues (0.14–1.20) and of the downregulated genes negative in all twelve (−1.17 to −0.13), with colon means of 0.28 and −0.67, whereas the fraction of individual genes shifting in the set direction ranged from 0.43 to 1.00 (Supplementary Table S16a,b).

The principal blocked voom-derived signature behaved differently. Its control-adjusted score was not associated with SenMayo (ρ = 0.056; β = 0.197, 95% CI −0.641 to 1.035; *p* = 0.620; Figure 2C,D) or Fridman (ρ = −0.061; *p* = 0.992). GLP1R transcripts occurred in 28 reconstructed cells and no reconstructed macrophages (Supplementary Table S11d). Sparse transcript detection does not exclude receptor protein or indirect systemic effects.

### 3.4. Virtual IL1B Perturbations Predict Opposing State Shifts

**Figure 4:**
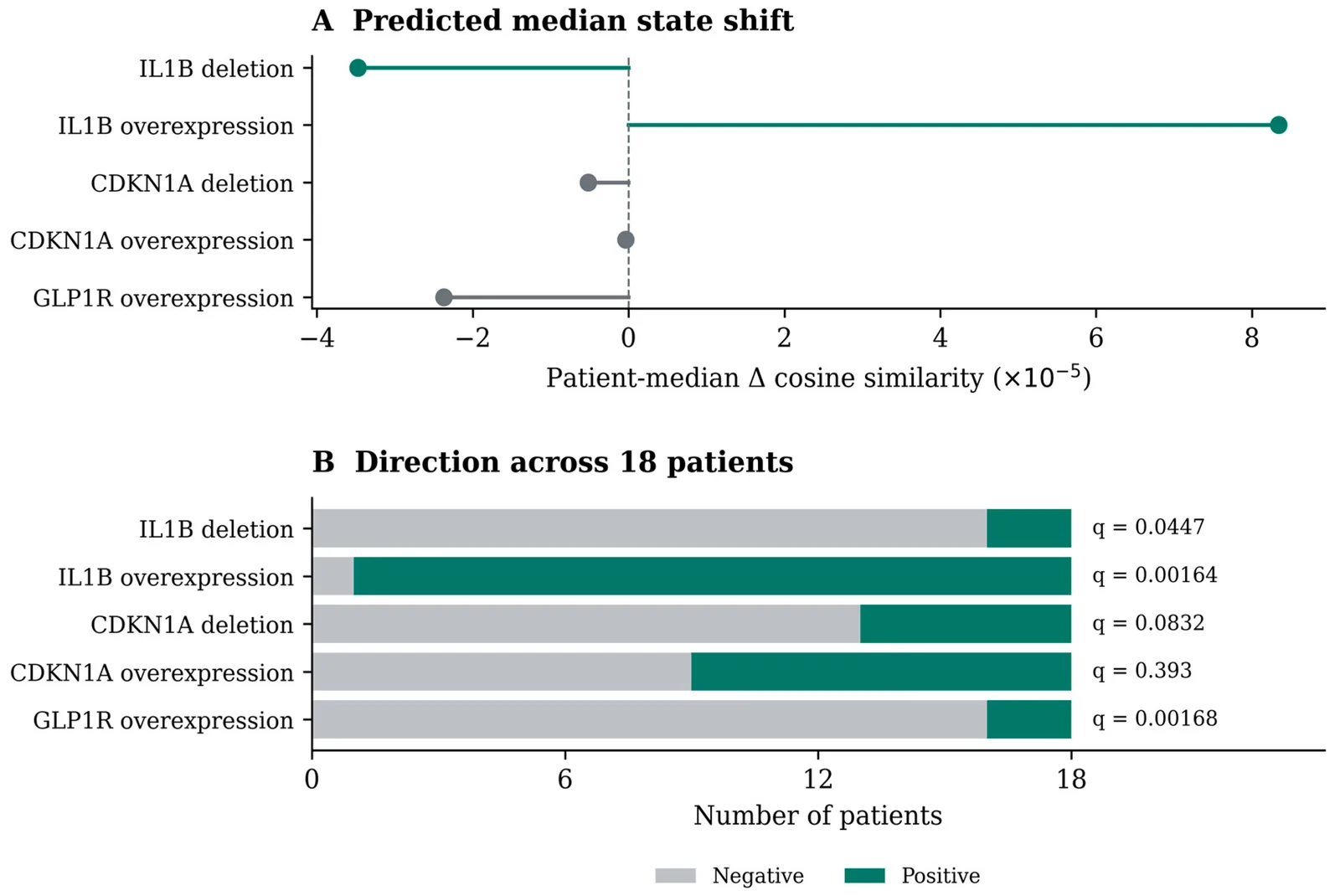
Original Geneformer perturbation predictions. (**A**) Median patient directional shifts from the original aggregate results. Positive values indicate a relative shift toward the senescence-high centroid. (**B**) Numbers of negative and positive patient shifts—q values apply BH correction across all five nominal patient-level Wilcoxon tests. Every arm includes 18 patients. IL1B deletion used 353 eligible cells, CDKN1A deletion used 226, and overexpression arms used 802 intermediate cells. Lines in (**A**) connect each estimate to zero and are not uncertainty intervals. In (**A**), Teal highlights the two IL1B perturbation arms, and the remaining perturbations are shown in gray. (Teal, positive/significant; gray, negative/non-significant)Predictions are conditional on the original state representation and do not constitute validation of the re-derived signature or experimental perturbation effects.

In the original Geneformer analysis, IL1B overexpression produced a median patient shift of +8.34 × 10^−5^ toward the high-score centroid—17 of 18 patients had positive shifts. Deletion produced a median shift of −3.47 × 10^−5^, with negative shifts in 16 of 18 patients. The five-perturbation BH q values were 0.00164 for overexpression and 0.0447 for deletion (Figure 4; Supplementary Tables S5d and S9).

Overexpression involved 802 intermediate cells, whereas deletion involved 353 cells containing the IL1B token. The two perturbations, therefore, did not use identical cell populations. CDKN1A deletion and overexpression were not significant after correction (q = 0.0832 and 0.393). GLP1R overexpression predicted a negative shift (q = 0.00168) but was a counterfactual in macrophages without detected receptor transcripts. These outputs describe model-dependent state shifts and do not establish experimentally measured senescence or treatment effects.

### 3.5. Recomputed Treatment Comparisons Do Not Show a Uniform Macrophage Response

Patient-level pseudobulk analysis identified 215 differentially expressed genes between high- and low-SenMayo tumor macrophages, including 124 upregulated and 91 downregulated genes (Supplementary Table S12b). Correctly labeled liraglutide analysis yielded no individual gene at FDR < 0.05, whereas semaglutide analysis yielded 594. These gene-level results were used to build treatment-ranked comparisons without selecting a favorable direction.

**Figure 5:**
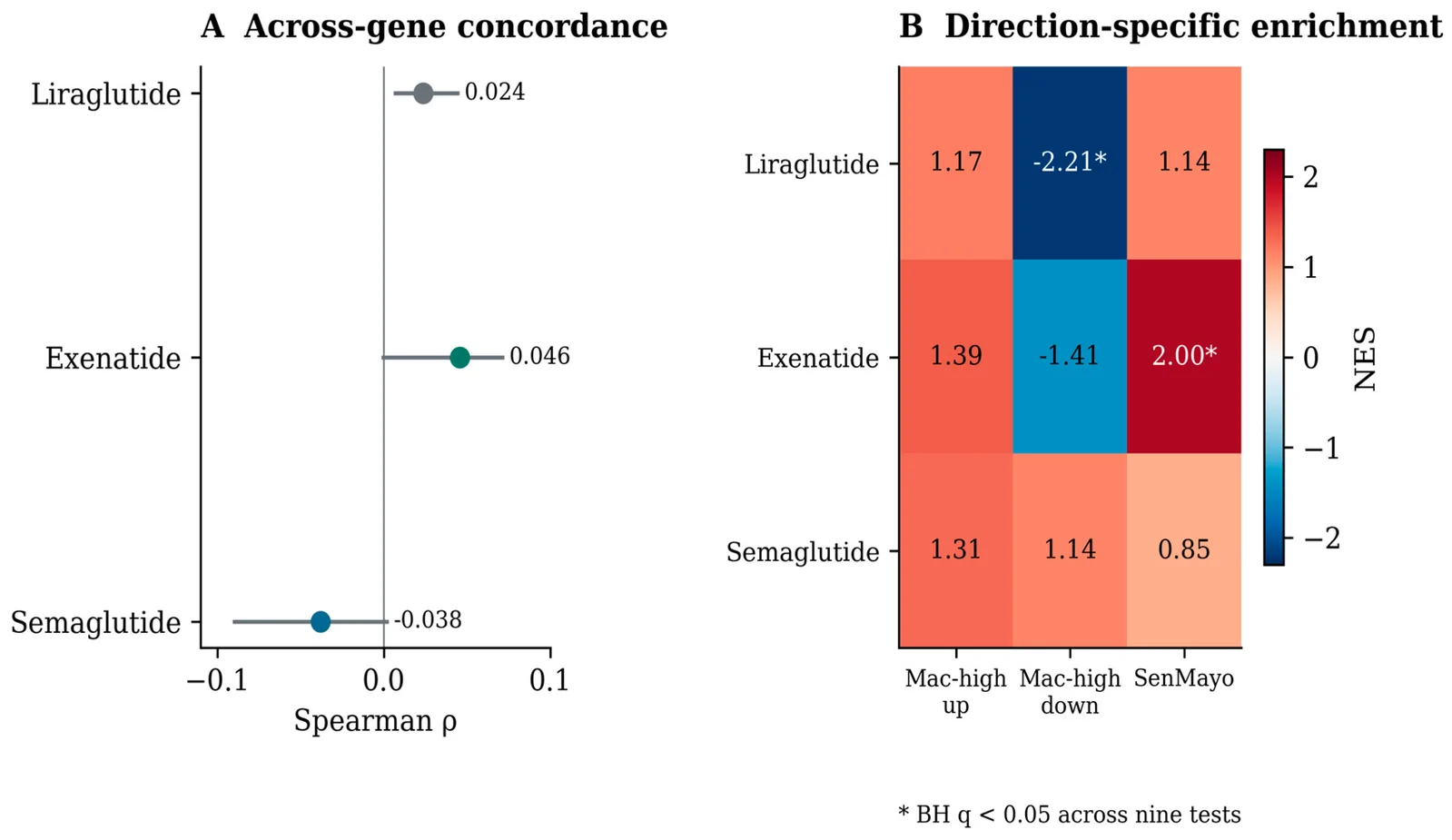
Recomputed murine treatment comparisons. (**A**) Spearman correlations between patient-level macrophage high-versus-low log fold changes and treatment-versus-control log fold changes over one-to-one mapped genes: 9120 for liraglutide, 9278 for exenatide, and 8689 for semaglutide. (**B**) Multilevel preranked enrichment NES values for macrophage high-state upregulated genes, high-state downregulated genes, and SenMayo. Asterisks indicate BH q < 0.05 across all nine tests. Macrophage directional sets required FDR < 0.05 and the stated fold-change direction. Positive enrichment of the downregulated set does not have the same interpretation as positive enrichment of the upregulated set. Liraglutide sample groups use verified GEO library identifiers, and exenatide uses the blocked voom model and also supplied the response signatures. In (**A**), points show full-sample estimates and lines show the minimum–maximum of leave-one-patient-out refits (Supplementary Table S15)—these ranges are not confidence intervals.

Across-gene fold-change correlations were small: ρ = 0.024 for liraglutide over 9120 genes, 0.046 for exenatide over 9278 genes, and −0.038 for semaglutide over 8689 genes (Figure 5A; Supplementary Table S7a). None indicates close transcriptome-wide correspondence with the macrophage high-score state. Leave-one-patient-out refits of the pseudobulk model showed that these small correlations were fragile at the patient level: the liraglutide correlation remained positive across refits (ρ = 0.006–0.045), whereas the exenatide and semaglutide correlations crossed zero in individual refits (ρ = −0.002 to 0.072 and −0.091 to 0.003; Supplementary Table S15), reinforcing their interpretation as weak and non-uniform.

Direction-specific enrichment was heterogeneous (Figure 5B; Supplementary Table S7b). Liraglutide negatively enriched the macrophage high-state downregulated set (NES = −2.21; nine-test BH q = 5.70 × 10^−6^), while its high-state upregulated and SenMayo enrichments were not significant. Exenatide positively enriched SenMayo (NES = 2.00; q = 5.10 × 10^−5^), but neither macrophage directional set passed the nine-test threshold. Semaglutide showed no enrichment surviving that correction. These comparisons involve different mouse tissues and experimental contexts. The exenatide dataset also supplied the response signatures, precluding an independent pharmacological validation claim.

## 4. Discussion

The original exenatide signature captures a macrophage expression pattern associated with senescence scores that was retained in several post hoc sensitivity analyses within the same cohort. The association was present in the original patient aggregates and in a reconstruction using a separate public cell-selection list and explicit marker rules—these are complementary analyses of the same colorectal cancer cohort (18 patients; 17 eligible for tumor-level reconstruction) rather than independent replication. It persisted after removing CXCR2 and IL1B from both programs and exceeded most expression-matched random signatures. These analyses strengthen the evidence for coordinated expression beyond direct gene overlap.

That result does not make the signature a robust pharmacological biomarker. Re-derivation changed its membership, and the resulting macrophage associations depended on the treatment-expression model. Blocked log-expression models retained strong associations, while blocked voom models did not. Both account for mouse-level dependence, so the discrepancy cannot be summarized as an effect of correcting repeated measures alone. Precision weighting across heterogeneous tissues, library-size handling, gene-selection thresholds, and orthologue mapping all contribute to how a treatment-associated program is represented. The appropriate conclusion is model sensitivity, with the original association retained as a biological observation.

The original upregulated genes include inflammatory and blood-cell-associated transcripts. Their coordinated expression in macrophages can plausibly connect the response score to SenMayo without indicating cellular senescence in a functional sense. Removing shared genes addresses direct arithmetic overlap, but it does not remove co-regulation or distinguish stable cell-cycle arrest from inflammatory activation. This ambiguity has biological anchors: GLP-1 receptor agonists attenuate macrophage inflammatory activation [16], senescent macrophage states have been described in tumors [19], and in colorectal cancer macrophage-derived IL1B can induce senescence in cancer-associated fibroblasts [20]. A treatment-associated pattern overlapping senescence scores in macrophages is, therefore, plausible without implying drug-induced senescence. The random-signature analysis likewise compares expression-matched genes, not every competing inflammatory or activation program. Complementary functional measurements remain necessary to establish the phenotype represented by the scores. The multi-tissue derivation estimates a shared systemic treatment-associated program rather than a tissue-specific response: mean treatment effects of the signature gene sets were directionally consistent across the twelve sampled tissues, while the macrophage association depended on the contributing tissue mix. This tissue composition dependence further limits a pharmacological reading of the response score.

The virtual perturbation results are consistent with an IL1B-related hypothesis within the original embedding model. Opposing deletion and overexpression predictions are more informative than an unsigned network response, but the perturbed cell populations differ, and the centroids derive from the same patients. Their very small embedding changes have no established conversion to biological effect size. Foundation model benchmarks further caution against assuming that plausible predictions demonstrate the mechanism [13,14]. These findings do not establish that IL1B mediates an exenatide effect or that receptor overexpression reproduces drug exposure.

Recomputed drug comparisons also argue for contextual interpretation. The weak fold-change correlations and differing enrichment patterns did not identify a uniform class-wide macrophage response. Positive SenMayo enrichment in the exenatide dataset is a tissue-level treatment observation—it does not demonstrate senescence induction in human colorectal macrophages. Conversely, lack of significance for the re-derived human score is not proof that exenatide has no macrophage effects. Exposure-defined human cohorts and direct experiments are required for either conclusion.

Several limitations remain intrinsic to the study design. The human data are observational, the colorectal cohort contains 18 patients, and reconstruction leaves fewer evaluable macrophage pairs. The two preprocessing representations are not independent cohorts. Marker-based annotation can misclassify cells, although the stricter definition supported the original tumor-level association. The multi-tissue treatment models estimate an additive average and may obscure tissue-specific responses. Post hoc sensitivity analyses and candidate perturbations do not provide prospective validation. Archived bulk coefficients were available for numerical inspection, but bulk preprocessing was not repeated in this reconstruction. The archived pan-cancer family included twelve tumor types, and the non-gastrointestinal cohorts, including breast, were reported here only as retained archived coefficients rather than as tested hypotheses.

The inflammation-adjustment analyses make this concern explicit: adjustment for either inflammatory score attenuated the association, and the broader inflammatory response score shares 25 of its 200 genes with SenMayo, so the two programs are not separable at the transcript level in these data. Specificity for cellular senescence over inflammatory activation, therefore, cannot be established—the macrophage pattern is interpreted as an inflammatory state that also carries senescence score information. A focused experimental follow-up would compare exenatide exposure, IL1B manipulation, and matched inflammatory stimulation in human macrophages. Receptor protein measurements, proliferative capacity or stable arrest, and complementary senescence markers would help distinguish inflammatory activation from senescence. The current analysis identifies a transcriptional pattern retained in several post hoc sensitivity analyses within the same cohort and defines the methodological limits that should guide those experiments.

## 5. Conclusions

The original exenatide-associated signature correlates with colorectal macrophage senescence scores beyond two shared genes and expression-matched random signatures. Its association is sensitive to treatment signature derivation, and murine drug comparisons do not support a uniform pharmacological interpretation. The findings prioritize inflammatory macrophage states for experimental study while retaining a clear distinction between transcriptional resemblance, predicted perturbation effects, and measured treatment biology.

## Figures and Tables

**Table 1:** Public datasets used in this study, their available analysis scale, and their role and interpretation.

**Dataset**	**Available Analysis Scale**	**Role and Interpretation**
Toil TCGA/GTEx	7101 discovery samples; 13 cancer types	12 complete-case bulk SenMayo models
GSE294300 original representation	278,083 cells; 8240 tumor macrophages	18 tumor patients; 17 macrophage pairs
GSE294300 public reconstruction	209,207 cells; 4316 tumor macrophages	17 eligible tumor patients; 10 macrophage pairs; same source cohort
GSE280382 AgedLT	93 treated/102 control tissue samples; 8/9 mice	12 tissues; original and re-derived signatures
GSE302656	5 treated/5 control mice	Liraglutide; lung tumors; verified library-to-group map
GSE338091	5 treated/5 control mice	Semaglutide; aged liver

## Data Availability

The source data are publicly accessible through GEO (GSE280382, GSE294300, GSE302656, and GSE338091) and the UCSC Xena Toil hub. Source URLs, retrieval dates, and checksums are given in Supplementary Table S1. The Supplementary Material additionally provides the exact gene sets and their checksums (Supplementary Table S2a–c), sample maps and verified group assignments (Supplementary Tables S1b,c and S12a), exact expression-matched control-gene lists and scoring gene lists, per-sample reconstruction quality metrics (Supplementary Table S11a), reconstructed patient and cell scores (Supplementary Table S11b,c), gene-level reconstructed macrophage expression, treatment comparison gene-level results, and all analysis scripts. Public raw matrices can be retrieved using the included download scripts.

## Supplementary Materials

The following supporting information can be downloaded at: https://www.mdpi.com/article/10.3390/genes17101266/s1. Supplementary Table S1a,b provide public data URLs, retrieval dates, file sizes, and checksums, together with the GSE280382 sample metadata and its provenance notes (S1c). Table S2a–c give the reconstructed and re-derived gene memberships, their checksums, and provenance notes. Table S3a–c report archived bulk associations, response-score survival models, and descriptive score correlations. Table S4 gives original patient–tissue–lineage scores. Table S5 gives the original aggregate virtual perturbation predictions. Table S6 gives the reproduced age- and sex-adjusted lineage models. Table S7a,b give the recomputed treatment correlations and direction-specific enrichment. Table S8a–c give original patient influence, paired-regression, and bootstrap checks. Table S9 gives the multiplicity correction across the five virtual perturbation tests. Table S10a,b give the signature derivation summary and within-mouse correlation estimates. Table S11a–d give reconstructed per-sample counts, patient scores, all 48 distinct models, and gene coverage. Table S12a,b give the verified liraglutide sample map and the complete macrophage pseudobulk differential expression. Table S13a,b give the derivation and mapping sensitivity results and the 1000 expression-matched random-signature correlations. Supplementary Figure S1 shows the random-signature distribution. Supplementary Data provide the analysis scripts, exact gene sets, exact expression-matched control-gene lists, environment records, and gene-level result tables. Table S14 gives per-patient inflammatory activation scores (S14a) and inflammation-adjustment association models (S14b). Table S15 gives leave-one-patient-out refits of the across-gene treatment correlations. Table S16 gives leave-one-tissue-out signature re-derivations and macrophage associations (S16a) and tissue-specific treatment effects for the original signature genes (S16b). Table S14b reports full and gene-disjointed inflammation scores with model-specific degrees of freedom, while Table S16a reports both full-set and two-sided gene-disjointed scoring. Only sub-tables cited in the text are included in the Supplementary Materials.

## Abbreviations

BH, Benjamini–Hochberg; BRCA, breast invasive carcinoma; COAD, colon adenocarcinoma; CRC, colorectal cancer; FDR, false discovery rate; GEO, Gene Expression Omnibus; GLP1R, glucagon-like peptide-1 receptor; GTEx, Genotype–Tissue Expression project; MGI, Mouse Genome Informatics; MSigDB, Molecular Signatures Database; NES, normalized enrichment score; READ, rectal adenocarcinoma; STAD, stomach adenocarcinoma; TCGA, The Cancer Genome Atlas; TMM, trimmed mean of M values.
